# What is the emotional core of the multidimensional Machiavellian personality trait?

**DOI:** 10.3389/fpsyg.2013.00454

**Published:** 2013-07-22

**Authors:** Syrina Al Aïn, Arnaud Carré, Carole Fantini-Hauwel, Jean-Yves Baudouin, Chrystel Besche-Richard

**Affiliations:** ^1^Developmental Ethology and Cognitive Psychology Group, Center for Smell, Taste, and Food Science, UMR 6265, CNRS and 1324 INRA, Université de BourgogneDijon, France; ^2^Cognition, Santé, Socialisation (C2S) EA 6291, Université de Reims Champagne-ArdenneReims, France; ^3^Faculté des sciences psychologiques et de l'éducation Unité de psychologie clinique et différentielle, Université libre de BruxellesBruxelles, Belgium; ^4^Institut Universitaire de FranceParis, France

**Keywords:** Machiavellianism, alexithymia, empathy, theory of mind, anhedonia, anxiety

## Abstract

Machiavellianism is a personality trait characterized by interpersonal manipulation and associated with specific patterns of emotional and social cognition skills. The aim of this study was to investigate its socio-cognitive characteristics by determining its association and predictors on the basis of a multidimensional approach to Machiavellianism. We used Mach IV scale to assess “Machiavellian Intelligence” skill of participants (Christie and Geis, 1970). It includes three subscales that are (1) the use of deceit in interpersonal relationships, (2) a cynical view of human nature and (3) the lack of morality. Associations were found between Machiavellianism and low levels of empathy and affective ToM, and high levels of alexithymia, anhedonia, depression, and anxiety. These associations were observed in varying proportions depending on the three subscales of Machiavellianism. The addition of anhedonia and trait-anxiety to the concepts of empathy and alexithymia made it possible to gain a better understanding of the emotional core of Machiavellianism. These findings are discussed in the light of developmental and adaptive perspectives.

## Introduction

Machiavellianism is a concept that has been accorded a growing level of interest, especially in the field of personality studies (Rauthmann, 2012). People exhibiting high levels of Machiavellianism (Christie and Geis, 1970) are characterized by interpersonal manipulation, such as the use of flattery and deceit, as well as by aloof, cynical, and traditionally amoral viewpoints adopted in order to promote their own goals/interests (Christie and Geis, 1970; Fehr et al., 1992; McHoskey, 1995; McHoskey et al., 1998; Wilson et al., 1998; Bereczkei et al., 2010). The Mach-IV scale assesses “Machiavellian Intelligence” competence of participants (Christie and Geis, 1970). This is a self-report Likert type scale which evaluates three dimensions: (1) the use of deceit in interpersonal relationships (nine items), (2) a cynical view of human nature (nine items), and (3) the lack of morality (two items).

It seems natural to assume that Machiavellian individuals can easily read the minds of others and understand social situations (Davies and Stone, 2003; Czibor and Bereczkei, 2012) which they can successfully manipulate in the service of their own intrinsic motivations (Fehr et al., 1992; Jones and Paulhus, 2009). Consequently, a number of questions have been raised concerning Machiavellian abilities and their determinants. For example, studies have revealed a non-significant link between this trait and general intelligence. It has been suggested that this is due to the specificity of the processes involved in Machiavellianism rather than to general skills (i.e., such as total IQ) (Wilson et al., 1996; Paulhus and Williams, 2002; Jones and Paulhus, 2009).

It has also been suggested that Machiavellian individuals have better “mind-reading skills” or Theory of mind (ToM) (Davies and Stone, 2003). In addition, Machiavellian individuals have frequently been characterized in terms of their detachment and lack of emotional involvement with others (Wrightsman, 1991). Their emotional disconnection seems to be similar to that observed in two emotional deficits: alexithymia and anhedonia. In addition, the same emotional detachment has been observed in people suffering from schizophrenia, depression and anxiety disorders (Mennin et al., 2009; Demenescu et al., 2010). However, the relationship between Machiavellianism and emotional deficits has been found to vary depending on the disorders studied and, at the same time, the various studies have investigated only a small number of disorders, focusing primarily on depressive and anxious disorders.

The question of the nature of what it is that lies at the heart of the emotional impairments associated with the presence of Machiavellian behaviors in a healthy population was addressed in the study reported here in an endeavor to gain a better understanding of the characteristics of this multidimensional concept. The debate on determination of emotional characteristics of psychopathology is here applied to the Machiavellianism in a dimensional way. We suggest that the infra-clinical approach could help to take into account the facets of Machiavellian behaviors and differentiated the cognitive and affective components that participate to explain the base of this behavior and its multidimensional expression.

### Machiavellianism, theory of mind, and empathy

First of all, the empirical data concerning the relation between Machiavellianism and mindreading are subject to some debate (see Paal and Bereczkei, 2007; Lyons et al., 2010). The ability to deceive others has been positively associated with performances on ToM tasks in childhood when false belief tasks were used (Chandler et al., 1989; Russell et al., 1991) or negatively associated when a faux-pas task was used (Barlow et al., 2010). In the case of adults, the results are mixed: Paal and Bereczkei (2007) found no correlation between Machiavellianism and ToM [using a comprehension tasks that requires mindreading at different levels of intentionality (Kinderman et al., 1998)], unlike Lyons et al. (2010) who found a negative correlation between these variables [The Imposing Memory Task (IMT) (Kinderman et al., 1998; Stiller and Dunbar, 2007)]. Furthermore, some studies have suggested that Machiavellians are excellent mindreaders (Sutton, 2001; Davies and Stone, 2003; Esperger and Bereczkei, 2012). In addition, the apparent emotional deficit in Machiavellian individuals may be indicative of an inability to feel empathy. We know that Machiavellian individuals exhibit deficits in certain components of emotional intelligence such as emotion recognition and empathy (see Jones and Paulhus, 2009). Empathy consists in adopting another person's point of view in order to share and understand other people's emotions or attribute emotions to them. This also implies that a distance is maintained between the two speakers in order to avoid confusion between one's own personal feelings/emotions and those experienced by the other person (Ruby and Decety, 2004). At least the following two components of empathy have been identified: cognitive empathy (i.e., recognizing and understanding the emotional states of others) and affective empathy (i.e., sharing the emotional states of others, also referred to as emotional contagion) (Jolliffe and Farrington, 2004). Numerous studies have shown a negative correlation between empathy and Machiavellianism scores (Watson et al., 1994; Wastell and Booth, 2003; Austin et al., 2007). However, the results concerning the link between Machiavellianism and the components of empathy (such as affective and cognitive empathy) are still ambiguous. Some studies have suggested that Machiavellians are deficient only at the level of affective empathy (sharing of emotions), whereas their cognitive empathy (recognizing and understanding other people's emotions using “Reading the Mind in the Eyes” ToM Test) is intact, even high [Barnett and Thompson, 1985, quoted in McIllwain (2003); Repacholi et al. (2003); Richell et al. (2003)]. Conversely, other studies have reported that Machiavellians are also deficient in cognitive empathy and are less able to recognize the emotions of others, thus indicating that they may not be aware of the consequences of their acts [Reading the mind in the eyes test (Baron-Cohen et al., 2001; Lyons et al., 2010); facial emotion decoding task: (Simon et al., 1990); IRI scale: Laura, 2002, quoted in McIllwain, 2003]. To summarize, the relationship between Machiavellianism, affective empathy and cognitive empathy is still unclear and controversial.

### Machiavellianism and emotional deficits

Secondly, Machiavellian individuals might be characterized by a deficit in feeling and identifying their own emotions, such as alexithymia, or by an inability to experience pleasure, such as anhedonia, i.e., deficits that are relatively common in both clinical and non-clinical populations. Alexithymia is defined as the absence of the words required to express emotions and feelings (Sifneos, 1973). In addition, people with alexithymia have (1) difficulties in identifying their emotions/feelings, (2) difficulties in distinguishing their emotional state from their physical or physiological state, (3) limited imagination and creative abilities, (4) a lack of introspective capabilities (Krystal, 1987; Taylor, 2000; Berthoz et al., 2011; Gumley, 2011). It therefore comes as no surprise that healthy alexithymic individuals have been found to obtain high Machiavellianism scores (Simon et al., 1990; Wastell and Booth, 2003; Loas et al., 2007) since Machiavellian individuals have a dysfunctional connection to their own emotions. Wastell and Booth (2003) found that Machiavellianism and alexithymia are highly correlated, especially with regard to two components of alexithymia (assessed using the Toronto Alexithymia Scale, TAS): externally orientated thinking (EOT) and difficulties in identifying feelings (DIF). To date, this model of the link between Machiavellianism and alexithymia has led researchers to adopt the concept of “volitional Machiavellianism” in which alexithymia causes the lack of empathy (Wastell and Booth, 2003). The results obtained by Loas et al. (2007) support the view that alexithymia is interlinked with Machiavellianism, in particular at the level of the cynical view of human nature it entails. Moreover, Loas et al. suggested that the association between other dimensions, such as depression or affective blunting (i.e., anhedonia), with Machiavellianism should also be studied.

Indeed, the concept of Machiavellian personality is dominated by emotional detachment from others and a lack of interpersonal warmth, a description close to that of alexithymic individuals (Geis, 1978; Fantini-Hauwel et al., 2012). Furthermore, a second emotional deficit, known as anhedonia, has been defined as the inability to feel pleasure (Ribot, 1896) accompanied by the presence of a diminished sensitivity to aversive events (Hardy et al., 1986). These emotional deficits are frequent in depression and anxiety disorders (Treadway and Zald, 2011). Anhedonia is also considered to be factor of vulnerability or a defense mechanism (Chapman et al., 1982; Akiskal, 1992), which could also be related to Machiavellianism. Nevertheless, to our knowledge, no study has investigated the relationship between anhedonia and Machiavellianism.

As far as depression and anxiety are concerned, only a small number of studies have investigated the relationship between clinical and non-clinical depression and Machiavellianism and the results are ambiguous. In a geriatric population, depressed and non-depressed males did not differ in terms of Machiavellianism, whereas depressed females tended to be more Machiavellian than non-depressed females (LaTorre and McLeoad, 1978). Examining a student population at a Military Medical School, Bakir et al. (1996) found a positive association between Machiavellianism and depression in men only. In contrast, Skinner (1982) found no difference between Machiavellianism scores and depression. With regard to anxiety disorders, various older studies have reported a moderate positive correlation between anxiety and Machiavellianism (Jones et al., 1979; Nigro and Galli, 1985; Poderico, 1987), whereas a recent study found no link between these two dimensions (Ali et al., 2009). In sum, the relationship between anxiety and Machiavellianism has been viewed as counterintuitive and it is unclear how this paradox should be interpreted given that both positive (Ramanaiah et al., 1994; Jakobwitz and Egan, 2005) and non-significant correlations (Allsopp et al., 1991; Paulhus and Williams, 2002) have been found. Trait-anxiety is often considered as a unitary concept which underlies the predisposition to react in an anxious way (Spielberger, 1972; Rudaizky et al., 2012). At the same, one could also hypothesize that anxiety, and especially trait-anxiety, takes the form of clusters of negative affects beyond a simple association of anxiety with the emotion of fear (Bados et al., 2010; Corr, 2011).

Moreover, trait-anxiety could be seen as an awareness of negative contexts and consequences. Indeed, Spitzer et al. (2007) pointed out that the neural correlates of social norm compliance (i.e., prefrontal and orbital cortex, caudate nucleus) are mobilized more in punishment than in other situations. In addition, participants with high Machiavellianism scores have been found to mobilize this neural network more when faced with a risk of repression, and correlations with the activity of the orbital cortex are particularly high (Spitzer et al., 2007). We hypothesized that the fact that Machiavellianism appears to be motivated more by a fear of repression than by moral factors could be related to the higher trait-anxiety scores.

To date, only a small number of studies have investigated the correlation between Machiavellianism and emotional impairments, and it is possible that this factor could explain the social withdrawal/maladjustment observed in depressed and anxious individuals.

### Issues addressed and hypotheses

The primary aim of the present study was to examine, in a non-clinical population, the association between Machiavellianism, affective ToM, affective and cognitive empathy, emotional deficits—alexithymia, anhedonia, depression and anxiety—in a young, general, non-clinical population.

We assumed that the Machiavellianism score would correlate negatively with the cognitive and affective empathy scores and affective ToM, but positively with levels of alexithymia, depression, anxiety and anhedonia.

In order to better understand Machiavellianism, we explored the emotional features of each subdimension of the Mach-IV scale.

## Method

### Participants

One hundred and seven native French participants (63 females), recruited from the general population volunteered to take part in the study. Participants were recruited via opportunity sampling from the French general public and students from the University of Reims and Paris Ouest Nanterre la Défense (students randomly solicited on the University and personal acquaintances). They were tested in their homes and classrooms in University campus. They were aged between 18 and 30 years, with a mean age of 23.9 years (*SD* = 3.4). These participants had all obtained at least a High School diploma. Participants were excluded if they reported any history of head injury, neurological disease, or psychiatric disorders. These data were collected by an in-house questionnaire, and participants had to fill in past or present disorders. All successfully completed all the items in all the questionnaires. Informed consent was obtained and the study was designed in accordance with the Declaration of Helsinki.

### Materials

#### Self-report measures

***Mach IV.*** Machiavellianism was assessed using the Mach-IV scale [Christie and Geis, 1970, translation into French by Romney (1979)]. This scale contains 20 items which cover three dimensions: (1) the use of deceit in interpersonal relationships (nine items), (2) a cynical view of human nature (nine items) and (3) the lack of morality (two items). The participants indicated their degree of agreement with these statements on a seven-grade Likert-type scale (1 = strongly disagree; 7 = strongly agree) and the scores ranged from 20 (Low level of Machiavellianism) to 140 (High of Machiavellianism). Cronbach's a in the current study was 0.69 for the Mach-IV total; 0.51 for the Mach-IV 1; 0.41 for the Mach-IV 2.

***Toronto Alexithymia Scale (TAS-20).*** The TAS-20 [(Bagby et al., 1994); French version by Loas et al. (2001)] is a 20-item self-report questionnaire that provides a measure of alexithymia on the basis of a three-factor structure: (1) Difficulty Identifying Feelings (DIF); (2) Difficulty Describing Feelings (DDF); (3) Externally Oriented Thinking (EOT). Participants are required to rate their degree of agreement with each item on a five-point Likert Scale from 1 (Strongly disagree) to 5 (Strongly agree). The score therefore ranges between 20 and 100. Cronbach's a in the current study was 0.78 for the TAS-20 total score, 0.68 for the TAS-20 DIF, 0.77 for the TAS-20 DDF, and 0.53 for the TAS-20 EOT.

***Snaith–Hamilton Pleasure Scale (SHAPS)***. The SHAPS (Snaith et al., 1995; French version by Loas et al., 1997) is a 14-item self-report questionnaire used to evaluate physical anhedonia (i.e., inability to experience pleasure). The hedonic physical capacity (i.e., leisure activities, eating and drink) were assessed on a four-point Likert scale (from “strongly disagree” to “strongly agree”). The score therefore ranges between 14 and 56. Cronbach's a in the current study was 0.73.

***Beck Depression Inventory—short form (BDI II—QD-2A).*** Depression was evaluated with the BDI II scale (QD-2A short form) (Beck et al., 1961; French version by Pichot et al., 1984) consisting of 13 items. Each item comprises four statements corresponding to 4 levels of growing intensity symptoms from 0 to 3. The total scores can vary from 0 to 39. Cronbach's a in the current study was 0.67.

***Spielberger State-Trait Anxiety Inventory (STAI).*** The STAI [Spielberger, 1983; French version by Gauthier and Bouchard (1993)] consists of two 20–item questionnaires: the trait and state anxiety scales (TAI and SAI). In this study, we used only items from the TAI (STAI form Y-2). Participants reported their level of agreement on a Likert-type scale with the statements: “almost never” (1) to “almost always” (4) and scores ranged from 20 to 80 points. Cronbach's a in the current study was 0.89.

***Basic Empathy Scale (BES).*** The BES scale (Jolliffe and Farrington, 2006; translated into French by D'Ambrosio et al., 2009) consists of a 20-item questionnaire, of which 9 items assess cognitive empathy and 11 items evaluate affective empathy. The responses were collected with statements on a five-grade Likert-type scale (from 1 to 5) and the total scores range between 20 (lack of empathy) and 100 (high level of empathy). Cronbach's a in the current study was 0.78 for the BES tot, 0.69 for the cognitive BES and 0.77 for the affective BES.

#### Reading the mind in the eyes test

***Eyes Test.*** The Eyes Test (Baron-Cohen et al., 2001) was used to assess the ability of individuals to identify and attribute emotions to others. Thirty-six photographs of the eye region of the face were shown and each photograph was associated with four complex mental state descriptors printed around the picture (one at each corner). One of these words (the target word) correctly identified the mental state of the person in the photograph, while the other three were included as foils. The participants were instructed to choose the word that best described what the person was thinking or feeling. This task is thought to measure cognitive empathy and “pure ToM” (Baron-Cohen et al., 1997) since the participants are required to attempt to put themselves into the mind of the person shown in the photograph, and attribute a relevant mental state to them. The test was scored by totaling the number of items (mental states) correctly identified by the participant. Each correct response scores a point, making the range of scores between 0–36.

### Procedure

The test session was conducted in small groups consisting of two to five participants. The tests were presented in the following order: The “Eyes Test,” BDI-II, BES, SHAPS, Mach-IV, STAI-Y-2, TAS-20. In the “Eyes Test,” the photographs were presented in the same order in all sessions. All the questionnaires were completed on paper. Regarding the Eyes Test, thirty-six photographs of the eye region of the face were shown using a computer but the answers were reported on paper.

### Statistical analyses

Averages are reported as means (with SD) (Table 1). Pearson correlations were performed to explore the association between variables. Statistical significance was set at two-tailed *p* < 0.05. Data were analyzed using the Statistica statistical package (version 9.0, Statsoft, France). First, we explored the correlations between the emotional/cognitive variables and Machiavellianism. Second, we analyzed the Machiavellianism total score and the three subscales as subdimensions assessed using the Mach-IV, and analyzed the predictors (i.e., affective and cognitive variables) that could indicate the core emotional composition of this particular personality.

**Table 1:** **Correlations amongst Machiavellianism, empathy, affective theory, and emotional deficits**.

	**Mean (*SD*)**	**Mach-IV total**	**Mach-IV 1**	**Mach-IV 2**	**Mach-IV 3**	**BES total**	**BES cognitive**	**BES affective**	**Eyes test total**	**TAS-20 total**	**TAS DIF**	**TAS DVE**	**TAS EOT**	**SHAPS total**	**BDI-II**	**STAI-Y-2**
**Mach-IV total**	71.59 (3.3)	1.00														
**Mach-IV-I**	33.13 (6.55)	0.84^*^	1.00													
**Mach-IV-II**	31.81 (5.91)	0.83^*^	0.41^*^	1.00												
**Mach-IV-III**	6.64 (1.7)	0.54^*^	0.27^*^	0.45^*^	1.00											
**BES total**	78.48 (7.75)	−0.40^*^	−0.35^*^	−0.30^*^	−0.25^*^	1.00										
**BES cognitive**	36.85 (3.5)	−0.37^*^	−0.23^*^	−0.39^*^	−0.25^*^	0.72^*^	1.00									
**BES affective**	41.62 (5.78)	−0.31^*^	−0.33^*^	**−0.17**	−0.21^*^	0.91^*^	0.36^*^	1.00								
**Eyes Test**	24.31 (2.43)	−0.25^*^	**−0.12**	−0.30^*^	**−0.14**	0.07	0.18	**−0.01**	1.00							
**TAS-20 total**	46.91 (2.43)	0.42^*^	0.32^*^	0.36^*^	0.32^*^	−0.28^*^	−0.43^*^	**−0,12**	−0.26^*^	1.00						
**TAS-DIF**	15.77 (4.79)	0.25^*^	0.16	0.23^*^	0.24^*^	0.02	**−0.17**	0.13	−0.19^*^	0.78^*^	1.00					
**TAS-DDF**	13.46 (4.41)	0.39^*^	0.30^*^	0.33^*^	0.28^*^	−0.33^*^	−0.39^*^	−0.20^*^	−0.26^*^	0.77^*^	0.45^*^	1.00				
**TAS-EOT**	17.69 (4)	0.27^*^	0.23^*^	0.22^*^	0.16	−0.33^*^	−0.38^*^	−0.21^*^	**−0.10**	0.59^*^	0.16	0.19^*^	1.00			
**SHAPS total**	22.6 (4.43)	0.40^*^	0.30^*^	0.38^*^	0.17	−0.37^*^	−0.36^*^	−0.28^*^	**−0.09**	0.22^*^	0.16	0.16	0.15	1.00		
**BDI-II**	3.91 (2.98)	0.34^*^	0.20^*^	0.36^*^	0.23^*^	**−0.12**	−0.24^*^	**−0.02**	**−0.16**	0.29^*^	0.30^*^	0.18	0.12	0.36^*^	1.00	
**STAI-Y-2**	43.14 (8.57)	0.32^*^	0.23^*^	0.31^*^	0.20^*^	0.07	**−0.13**	0.16	**−0.07**	0.29^*^	0.43^*^	0.19	**−0.01**	0.33^*^	0.63^*^	1.00

Intercorrelations, Bivariate Pearson correlation coefficients (n = 107; ^*^P < 0.05). Mach-IV, Mach-IV scale (Machiavellianism); Mach-IV-I, deceit in interpersonal relationships; Mach-IV-II, cynical view of human nature; Mach-IV-III, lack of morality; BES, Basic Empathy Scale total score; TAS-20, Toronto Alexithymia Scale; TAS-DIF, Difficulty Identifying Feelings; TAS-DDF, Difficulty Describing Feelings; TAS-EOT, Externally Oriented Thinking; SHAPS, Snaith–Hamilton Pleasure Scale (Anhedonia); BDI-II, Beck Depression Inventory; STAI-Y-2, Trait-anxiety. Bold values indicate significant correlations at

* p < 0.05.

## Results

Descriptive statistics for the emotional and cognitive dimensions for the overall sample are presented in Table 1. The scores are quite similar to those reported in other studies.

### Correlations

Intercorrelations between Machiavellianism, affective ToM, cognitive and affective empathy, alexithymia, anhedonia, depression and anxiety calculated using Bravais-Pearson correlations are shown in Table 1. Machiavellianism was significantly correlated with most of the variables, including negative correlations with empathy and affective ToM, and positive correlations with alexithymia, anhedonia, depression, and anxiety.

### Regressions

Step-wise multiple regression analyses were performed in order to determine which dimensions of emotional functioning best predict Machiavellianism. In fact, this kind of analysis led to identify for the total score of the Mach-IV and for each of the subscales the differential influence of the socio-emotional factors included in the study. Indeed, a regression analysis permits to highlight the relative importance of each predictor (i.e., SHAPS, TAS-DIF,…) and to determine the specific effect of each one because it takes into account the relations between the various predictors entered in the regression (Howell, 1998). We therefore computed three linear regressions using as dependent variable each subscale of the Mach-IV. We also processed an analysis with the total score of the scale. We chose a selection method with *F* > 4 and *p* < 0.05, that constituted a classical threshold enough restrictive. This permitted to ensure that the selected regressors are not overestimated and only significant variables are retained (Tenenhaus, 1996; Howell, 1998). The total Machiavellianism score appeared to be predicted by anhedonia and affective aspects of alexithymia (TAS-DDF).

The use of deceit (Mach-IV-I), a cynical view of humanity (Mach-IV-II) and lack of morality (Mach-IV-III) were predicted at different levels by anhedonia, difficulties in describing feelings (DDF) (DIF and DDF), cognitive or affective empathy and ToM (Eyes Test). This point emphasizes the fact that an examination of the dimensional aspects of Machiavellianism has made it possible to show that different combinations of socio-cognitive components are involved in Machiavellian traits.

The results are presented in detail in Table 2.

**Table 2:** **Predictions of the Mach-IV total score and subscales amongst empathy, affective theory and emotional deficits scores**.

**Variable**	***B***	**SE B**	***F***	**β**	***R***	***R*^**2**^**
***(FOR MACH-IV-TOTAL SCORE)***					0.52	0.27
Constant	40.16^**^	5.37	55.98			
TAS-DDF	0.86^**^	0.22	15.48	0.33^**^		
SHAPS tot.	0.88^**^	0.22	16.46	0.34^**^		
***(FOR MACH-IV-I SCORE)***					0.47	0.22
Constant	36.93^**^	5.18	50.83			
STAI-Y-2	0.19^**^	0.07	7.28	0.24^**^		
BES-affective	−0.37^**^	0.10	13.27	−0.33^**^		
TAS-DDF	0.28^*^	0.14	4.25	0.19^*^		
***(FOR MACH-IV-II SCORE)***					0.55	0.30
Constant	50.63^**^	8.20	38.17			
BDI-II	0.41^*^	0193	4.61	0.19^*^		
BES-cognitive	−0.38^*^	0.15	6.32	−0.23^*^		
Eyes Test	−0.51^*^	0.21	6.23	−0.21^*^		
SHAPS tot.	0.28^*^	0.12	4.90	0.21^*^		
***(FOR MACH-IV-III SCORE)***					0.28	0.08
Constant	5.20^**^	0.51	103.27			
TAS-DDF	0.11^**^	0.04	8.76	0.28^**^		

* p < 0.05;

** p < 0.01. Mach-IV, Mach-IV scale (Machiavellianism); Mach-IV-I, deceit in interpersonal relationships; Mach-IV-II, cynical view of human nature; Mach-IV-III, lack of morality; BES, Basic Empathy Scale total score; TAS-20, Toronto Alexithymia Scale; TAS-DDF, Difficulty Describing Feelings; SHAPS, Snaith–Hamilton Pleasure Scale (Anhedonia); BDI-II, Beck Depression Inventory; STAI-Y-2, Trait-anxiety.

## Discussion

The aim of this study was (1) to explore the emotional characteristics of Machiavellianism and (2) to determine which of these are predictive of the different dimensions of Machiavellianism. First of all, we found that Machiavellianism was negatively correlated with cognitive and affective empathy. A number of studies have demonstrated similar results (Barnett and Thompson, 1985; Watson et al., 1994; Wastell and Booth, 2003; Andrew et al., 2008; Ali and Chamorro-Premuzic, 2010). Consequently, people who are able to feel/share, recognize and understand other people's emotions might have a tendency to inhibit Machiavellian behavior. Difficulties in affective and cognitive empathy were differentially associated with the various dimensions of Machiavellianism. Indeed, affective empathy was a predictor of the use of deceit. A deficit in the cognitive component of empathy appeared to be a factor contributing to a cynical view of humanity. We observed a correlation between Machiavellianism (total score) and the capacity to identify complex emotions or feelings in the “Eyes Test.” More specifically, these difficulties in mindreading were associated with the cynical aspects of the trait. The results revealed differences between empathy and affective ToM assessments and consequently suggest a distinction between these two socio-cognitive concepts. The empathy scale corresponds to a self-report that measures an individual's own assessment of his/her ability to identify and understand primary emotions in others (i.e., happiness, sadness, anger), whereas the “Eyes Test” evaluates the ability to identify certain complex emotional states. Evidence in support of this interpretation can be found in the fact that the correlations between these two concepts were weak. Machiavellian individuals may exhibit difficulties in attributing complex emotions to others and find it difficult to understand and interpret affective states. This study could also provide additional evidence about the relevance and validity of the Eyes Task. Machiavellian dimensions appeared to be associated with and predicted by alexithymia (but see Vellante et al., in press). Indeed, the Machiavellian participants found it difficult to identify and describe their own feelings/emotions and were characterized by external thinking. The difficulty in describing feelings appeared as a predictor of the Machiavellianism and Machiavellian's traits (Mach-IV-I and Mach-IV-III). Machiavellian individuals seem to judge human nature more negatively and be more inclined to use duplicitous strategies. These findings are consistent with previous studies showing a correlation between alexithymia and Mach-IV (Wastell and Booth, 2003; Loas et al., 2007), as well as between alexithymia and empathy (Davies et al., 1998; Jolliffe and Farrington, 2006; D'Ambrosio et al., 2009; Carré et al., 2013).

The correlations between the externally oriented thinking (TAS-EOT) component of alexithymia and the Mach-IV total score or subscales could refer to a concrete and logical mode of thought which does not take account of emotional reactions. Nevertheless, the general Machiavellianism score appeared to be predicted by difficulties in describing feelings. This observation emphasizes the emotional impairments present in Machiavellian persons.

Second, anhedonia was also found to be positively associated with Machiavellianism and negatively associated with empathy: people who find it difficult to feel physical pleasure should therefore be more Machiavellian and less empathic (at the level of both cognitive and affective empathy). To date, no study has addressed and investigated this question. Increased levels of anhedonia were found to be associated with trait-anxiety as well as with difficulties in cognitive empathy, affective ToM and in describing feelings, and also seemed to be related to cynicism. This finding could be seen as proof of the emotional difficulties that lead to Machiavellian behaviors.

In addition, state-depression and trait-anxiety scores were also found to be positively correlated with Machiavellianism scores. In accordance with our hypothesis, the individuals with high scores for depression and/or anxiety symptoms obtained high Machiavellianism scores. Trait-anxiety, which reflects negative affectivity, was a predictor of the Mach-IV-I subscale. This result, which emphasizes the relationship between temperamental anxiety and Machiavellianism, could reflect an awareness of negative consequences. This point could help highlight differences between the “Machiavellian mind” and other personalities and disorders considered to be related to social maladjustment (Spitzer et al., 2007).

However, we have to be cautious about these interpretations as this study has used mainly self-questionnaires. Some participants might communicate, consciously or not, some information which is socially acceptable or consensual, instead of true information about their personality. Thereby, an assessment about the level of socially desirability could be controlled and more test measures could be used in this study (e.g., facial expressions, and physiological responses such as electromyography). In other words, people are prompted to perform Machiavellian actions in response to their unusual patterns of social cognition, and other assessments could be able to underline it in a stronger way. Despite this, they act as a function of the consequences they risk and of which they are aware. Their level of negative affectivity, which is underpinned by their trait anxiety, would lead them to act if the feedback and negative consequences were limited (Spitzer et al., 2007). Another possible limit concerns the dimensionality of the Mach-IV: Rauthmann (2013) noted that “the dimensionality of the Machiavellianism construct is also conceptually unclear” (p. 388). This author suggests to use a new short-version of the Mach-IV, the MACH^*^, based on five items exploring cynicism and misanthropy (Rauthmann, 2013). Finally, we suggest being cautious with the current analyses, and suggesting that further studies should be conducted with larger samples sizes and therefore with a better account of colinearity.

## Conclusions

To date, no study apart from the one reported here has used this type of tool to investigate the relationship between Machiavellianism and emotional deficits. The results show that there is a negative association between Machiavellianism and (affective/cognitive) empathy and affective ToM, and a positive association between Machiavellianism, levels of alexithymia, anhedonia, depression, and anxiety. The results of the predictions suggest that statistical tools should be used as part of a dimensional approach (i.e., regression) in addition to the category analysis tools.

Moreover, our results emphasize the role of these different emotional components in vulnerability to Machiavellian traits. Future research should investigate clinical and non-clinical populations suffering from depression, schizotypal personality disorders or schizophrenia in order to evaluate the potentially aggravating impact of emotional deficits on Machiavellianism. This type of study could lead to the development of accurate evaluation systems and appropriate therapeutic support and care as a function of the socio-emotional profile of the patient.

### Conflict of interest statement

The authors declare that the research was conducted in the absence of any commercial or financial relationships that could be construed as a potential conflict of interest.
